# Testing Bidirectional, Longitudinal Associations Between Disturbed Sleep and Depressive Symptoms in Children and Adolescents Using Cross-Lagged Models

**DOI:** 10.1001/jamanetworkopen.2022.27119

**Published:** 2022-08-22

**Authors:** Cecilia Marino, Brendan Andrade, Jacques Montplaisir, Dominique Petit, Evelyne Touchette, Hélène Paradis, Sylvana M. Côté, Richard E. Tremblay, Peter Szatmari, Michel Boivin

**Affiliations:** 1 Hospital for Sick Children, Toronto, Ontario, Canada; 2 Cundill Centre for Child and Youth Depression, Centre for Addiction and Mental Health, Toronto, Ontario, Canada; 3 Department of Psychiatry, Temerty Faculty of Medicine, University of Toronto, Toronto, Ontario, Canada; 4 Center for Advanced Research in Sleep Medicine, Centre Intégré Universitaire de Santé et de Services Sociaux du Nord-de-l’île-de-Montréal, Montreal, Québec, Canada; 5 Department of Psychiatry, Université de Montréal, Montreal, Québec, Canada; 6 Department of Psychoeducation, Université du Québec à Trois-Rivières, Trois- Rivières, Québec, Canada; 7 Research Unit on Children’s Psychosocial Maladjustment, Université du Québec à Trois-Rivières, Trois-Rivières, Québec, Canada; 8 Groupe de Recherche sur l’inadaptation Psychosociale chez l’enfant, Université Laval, Québec City, Québec, Canada; 9 Department of Psychology, Université de Montréal, Montreal, Québec, Canada; 10 School of Psychology, Université Laval, Québec City, Québec, Canada

## Abstract

**Importance:**

Understanding the longitudinal, bidirectional associations between disturbed sleep and depression in childhood and adolescence is crucial for the development of prevention and intervention programs.

**Objective:**

To test for bidirectional associations and cascade processes between disturbed sleep and depressive symptoms covering both childhood and adolescence and to test for the moderating processes of sex and pubertal status in adolescence.

**Design, Setting, and Participants:**

A prospective cohort study using the Québec Longitudinal Study of Child Development (QLSCD; 1997-ongoing). QLSCD’s objective is to identify early childhood factors associated with long-term psychosocial and academic adjustment. Data were collected across 8 waves between ages 5 years (2003) and 17 years (2015). Associations were tested through cross-lagged models in childhood (5, 7, and 8 years), and in adolescence (10, 12, 13, 15, and 17 years). Data were analyzed from February to October 2021.

**Main Outcomes and Measures:**

Primary outcomes were disturbed sleep and depressive symptoms. Disturbed sleep was parent-reported and included sleep duration, time awake in bed, daytime sleepiness, sleep talking, sleepwalking, night terrors, and nightmares. Depressive symptoms were parent-reported in childhood (Child Behavior Checklist and Revised Ontario Child Health Study Scales), and self-reported in adolescence (Mental Health and Social Inadaptation Assessment for Adolescents).

**Results:**

Data on 1689 children (852 female [50.4%]) and 1113 adolescents (595 female [53.5%]) were included in the analyses. In childhood, significant bidirectional associations between depressive symptoms and disturbed sleep at all time points were found, indicating cascade processes (range β = 0.07; 95% CI, 0.02-012 to β = 0.15; 95% CI, 0.10-0.19). In adolescence, significant bidirectional associations from depressive symptoms to disturbed sleep (β = 0.09; 95% CI, 0.04-0.14) and vice versa (β = 0.10; 95% CI, 0.04-0.16) between 10 and 12 years were found. Between 12 and 13 years, depressive symptoms were modestly associated with disturbed sleep (β = 0.05; 95% CI, 0.001-0.10) but the reverse association was not significant. Cross-lagged estimates were nonsignificant after 13 years. The associations did not vary as a function of either sex or puberty-by-sex.

**Conclusions and Relevance:**

These findings suggest that disturbed sleep is associated with the consolidation of depressive symptoms starting in childhood, which, in turn, is associated with ongoing sleep problems. It is possible that timely and appropriate interventions for incipient disturbed sleep and depression prevent spiraling effects on both domains.

## Key Points

**Question:**

What are the bidirectional, longitudinal associations between disturbed sleep and depressive symptoms in children and adolescents?

**Findings:**

In this cohort study including data on 1689 children and 1113 adolescents, cross-lagged analyses showed significant cascade processes (ie, bidirectional links carried forward across time) throughout childhood. In adolescence, significant bidirectional associations were found between ages 10 and 12 years, but after age 13 years, no significant cross-lagged estimates were found.

**Meaning:**

These findings suggest disturbed sleep in childhood is an important target for early identification and intervention programs for depression.

## Teaser

This cohort study evaluates the bidirectional, longitudinal associations between disturbed sleep and depressive symptoms in children and adolescents using cross-lagged models.

## Introduction

Depression is the third leading cause of disability worldwide.^1^ It is an all-age disorder, and childhood is the time when it can first appear.^2^ The cumulative prevalence is low in childhood (2%-4%) and increases sharply during adolescence, peaking around 20% in early adulthood.^3^

Although girls are affected as often as boys in childhood, girls show a higher risk (approximately 2:1) from adolescence onward.^4^ Among other risk factors,^4^ earlier puberty is associated with an increased risk of depression and with the preponderance of affected girls in the transition from childhood to adolescence.^5,6^ Longitudinal epidemiological studies show that individuals with child-onset or adolescent-onset depression (compared with adult-onset depression) have a more chronic and recurring course, show worse functional outcomes,^3,7,8^ and have poorer response to treatment,^9,10,11^ suggesting that focusing on early prevention is important and could be key to reducing the overall lifetime burden of depression.

Prevention programs for depression in children and adolescents that rely on cognitive, coping, and emotional management skills are effective.^12^ However, their effects tend to decay over time,^13,14^ suggesting that further effort is needed. Modifiable risk factor management has proven effective in the prevention of various diseases^15^ and may be a complementary strategy to bolster depression prevention programs.

Disturbed sleep is one of the many modifiable risk factors for depression in children and adolescents.^16,17^ Previous research used a wide range of definitions for disturbed sleep, including insomnia symptoms,^18,19,20,21,22,23^ chronotype,^24^ daytime sleepiness,^25^ and broadly defined sleep disturbances, which include both insomnia and parasomnias (ie, nightmares, sleep talking, sleepwalking, and night terrors).^26,27,28,29,30,31,32,33,34,35,36,37,38^ A recent meta-analysis^39^ showed that the pooled estimates of the association between disturbed sleep and depression did not differ significantly between insomnia and broadly defined sleep disturbances.

Children and adolescents often report both disturbed sleep and depression, with co-occurrence rates equally distributed between boys and girls.^40,41^ Significant longitudinal, unidirectional associations have also been reported. A recent meta-analysis^39^ reported significant pooled estimates of the association between disturbed sleep and later depression controlling for baseline depression. Treating disturbed sleep has been effective in decreasing later depression in adolescence.^42^ However, whether analog prevention approaches in childhood will result in similar, or enhanced, outcomes is still unknown. There is also evidence of the prospective, unidirectional association between depression and later disturbed sleep in children and adolescents,^31,43,44^ although this finding is mixed, with some studies reporting nonsignificant associations.^32,41,45^

Several studies focused on bidirectional associations between disturbed sleep and depression in children and adolescents,^46^ with a minority testing 3 or more time points, therefore potentially looking at developmental cascades. Cascade processes occur when bidirectional links are carried forward across time, above and beyond within-time covariation and within-construct continuity.^47^ As of this writing and to our knowledge, 6 studies^31,37,48,49,50,51^ have tested 3 or more time points, and developmental cascades were found in adolescence only.^48^ Only 2 studies^50,51^ focused on childhood, and no studies covered both childhood and adolescence, which hinders a full understanding of the dynamics at play throughout these developmental stages.

Understanding the longitudinal, bidirectional associations and possible developmental cascades between disturbed sleep and depression has the potential to inform risk identification and prevention programs. The fact that disturbed sleep may lead to depression and, potentially, vice-versa, can trigger an unhealthy cycle. If set in motion in childhood, such a cycle would have time to amplify, resulting in developmental cascades that consolidate the 2 problems (ie, reciprocal effects build up over time, with long-lasting cumulative consequences for development).^47^

The primary goal of the current study was to replicate and expand this previous research by testing bidirectional associations and developmental cascades between disturbed sleep and depression using cross-lagged models and by extending the developmental window of investigation to cover both childhood and adolescence. We used data from the Québec Longitudinal Study of Child Development (QLSCD) collected across 8 waves between ages 5 and 17 years. Given the differences in risk between child-onset and adolescent-onset depression, particularly for girls,^52^ and the different assessment tools used in these developmental periods, we analyzed childhood and adolescence separately. In addition, given sex-related and puberty-related prevalence differences in both depression and disturbed sleep in adolescence,^6,53^ we conducted 2 multigroup analyses to examine the possible moderating role of sex and puberty in the cross-lagged model in adolescence. According to previous literature,^31,37,49,51^ we expected no sex difference. Given the lack of prior literature, the second multigroup analysis was considered exploratory.

## Methods

### Sample

This cohort study follows the Strengthening the Reporting of Observational Studies in Epidemiology (STROBE) reporting guideline for observational studies.^54^ The QLSCD is a representative birth cohort of infants born between 1997 and 1998 to mothers living in the province of Québec, Canada. Attrition rate and detailed cohort characteristics are reported in eMethods 1 in the Supplement and elsewhere.^55^ The study was approved by the ethics review boards of the Institut de la Statistique du Québec, the Centre Hospitalier Universitaire Sainte-Justine, the Louis-Hippolyte Lafontaine Hospital, and the Université de Montréal, Faculty of Medicine, and written informed consent was obtained from families at each assessment.^55^

For childhood analyses, data collected at ages 5, 7, and 8 years were considered, whereas for adolescent analyses, data at 10, 12, 13, 15, and 17 years were used. Of note, in the current study, we considered childhood the period between 5 and 9 years of age, and adolescence the period between 10 and 17 years.^56^ To allow for comparability across studies, given that the literature varies considerably in the definition of childhood and adolescence cutoffs, we tested 3 additional cross-lagged models: (1) 5, 7, 8, and 10 years; (2) 12, 13, 15, and 17 years; and (3) 5, 7, 8, 10, 12, 13, 15, and 17 years. Participants were included if they had 2 or fewer missing values for depression or disturbed sleep across all time points. For childhood assessments, mothers were asked to report on their child’s sleep and depression, whereas for adolescence, mothers reported on their child’s sleep, and depression was self-reported by adolescents.

### Disturbed Sleep

For a wider scope, we opted for a broad definition of disturbed sleep, including both parasomnias and insomnia. Sleep was assessed through a questionnaire of 7 items that assessed sleep duration, time awake in bed, daytime sleepiness, sleep talking, sleepwalking, night terrors, and nightmares (eMethods 1 in the Supplement). The same measurement was used in previous research^57^ in the QLSCD cohort. A total score was computed, and the score was rescaled to range 0 to 10. The higher the score, the more disturbed the sleep. For comparability with previous research,^18,19,20,21,22,23^ cross-lagged analyses were repeated using an insomnia index—that is, the total score included sleep duration, time awake in bed, and daytime sleepiness.

### Depressive Symptoms

Parents rated children on the well-validated Behavior Questionnaire, which was created for the Canadian National Longitudinal Study of Children and Youth^58^ and incorporates items from the Child Behavior Checklist^59^ and the Revised Ontario Child Health Study Scales.^60^ Eight items were used to depict depressive symptoms (eMethods 2 in the Supplement). Adolescents completed a 10-item questionnaire drawn from the Mental Health and Social Inadaptation Assessment for Adolescents. This measure has been validated in the QLSCD^61^ (eMethods 3 in the Supplement). A mean score was computed, and the score rescaled to range 0 to 10. The higher the score, the more depressive symptoms.

### Covariates and Moderators

Sex, pubertal status, socioeconomic status (SES), and maternal depression were selected according to previous research.^5,6,34,62,63^ No data were available for sex. SES and maternal depression were entered as covariates at participant’s age 5 and 10 years in childhood and adolescence analyses, respectively. Sex and pubertal status were used as categorical moderators in multigroup analyses in adolescence. Measurements’ details for covariates and moderators are in eMethods 4 in the Supplement. Given that puberty-related risk for depression differs in boys and girls (ie, early pubertal status in girls, and early and late pubertal status in boys are associated with increased depression),^5^ pubertal status was categorized into 4 groups: early or late in boys/or girls (late = pubertal status lower than III; early = pubertal status equal or higher than III^64^).

### Statistical Analysis

Descriptive statistics and Pearson correlations were computed using SPSS statistical software version 19.0 for Windows (SPSS Inc.). Missing data were examined by the missing value analysis module and the Little Missing Completely at Random test.^65^ Cross-lagged models were conducted using Mplus statistical software version 7 (Muthén & Muthén).^66^ Missing data were treated through full information maximum likelihood, which uses maximum likelihood to estimate model parameters using all available raw data.^67,68^ Overall model fit was tested by considering together the comparative fit index (CFI), the Tucker-Lewis index (TLI), the root mean square error of approximation (RMSEA), the χ^2^ statistic and the ratio of χ^2^ to degrees of freedom.^69^ A nonsignificant χ^2^ value, a CFI and a TLI value of 0.90 or higher, an RMSEA value below 0.06, and a ratio of χ^2^ to degrees of freedom less than 3 were considered indicators of good fit.^70^ Secondary analyses used a multigroup approach to test whether model estimates differed across sex or puberty-by-sex using a χ^2^test. The unconstrained model was compared against a model in which across-time, within-time, and cross-lagged paths were constrained to be equal across levels of the moderator. A deterioration of model fit would suggest that the associations may be different across levels of the moderator. All *P* values were 2-sided. *P* < .05 was considered to be significant. Data were analyzed from February to October 2021.

## Results

A total of 1689 children (852 female [50.4%]) and 1113 adolescents (595 female [53.5%]) were included in the study. Missing data patterns for outcome variables in childhood and adolescence are reported in eResults 1, eTable 2, eTable 3, eTable 4, and eTable 5 in the Supplement.

### Childhood

In the childhood analysis, 1689 children (49.6% male) were included (Table 1). Participants were from a higher SES and were more often girls compared with nonparticipants (431 participants; *t*_2115_ = −6.55, *P* < .001; and χ^2^_1_ = 6.34, *P* = .011, respectively). Descriptive statistics across time points for the study variables are shown in Table 2.

**Table 1.** Characteristics of the Population Included in the Study

Characteristics	Participants, No. (%)	
	Childhood sample (n = 1689)	Adolescence sample (n = 1113)
Sex		
Female	852 (50.4)	595 (53.5)
Male	837 (49.6)	518 (46.5)
Low birth weight (<2500 g)	55 (3.3)	30 (2.7)
Prematurity (<37 wk of gestation)	84 (5.0)	45 (4.0)
Sociodemographic characteristics		
Parental age at child’s birth, mean (SD), y		
Mother	28.98 (5.18)	29.21 (5.09)
Father	31.82 (5.55)	31.89 (5.35)
Low education (no high school diploma)		
Maternal	244 (14.5)	139 (12.5)
Paternal	258 (16.5)	159 (15.2)
Nonintact family (single parent/blended)	296 (17.6)	183 (16.4)

**Table 2.** Descriptive Statistics in the Childhood Sample^a^

Time point and variable	Children, No. (N = 1689)	Total score, mean (SD) [range]
5 y		
Disturbed sleep	1438	1.50 (0.21) [0.00 to 10.00]
Depression	1632	2.33 (1.47) [0.00 to 10.00]
Maternal depression	1437	1.49 (1.69) [0.00 to 10.00]
Socioeconomic status	1689	0.04 (0.95) [−2.91 to 2.90]
6 y		
Disturbed sleep	1307	1.46 (0.20) [0.00 to 10.00]
Depression	1428	2.44 (1.54) [0.00 to 10.00]
8 y		
Disturbed sleep	1259	1.39 (0.20) [0.00 to 10.00]
Depression	1392	2.52 (1.58) [0.00 to 10.00]

^a^ Data are courtesy of the Quebec Institute of Statistics.

**Figure 1.**
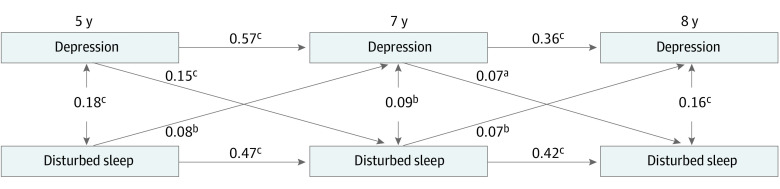
Estimates From the Cross-Lagged Model for the Reciprocal Associations Between Disturbed Sleep and Depression Across Childhood All estimates are standardized. Fit statistics: χ^2^_16_ = 52.449, *P* < .001; root mean square error of approximation = 0.037 (95% CI, 0.026-0.048); comparative fit index = 0.981; Tucker Lewis index = 0.961. Covariates were socioeconomic status and maternal depression. *P* < .05 was considered statistically significant. Data are courtesy of the Quebec Institute of Statistics. ^a^*P* < .05. ^b^*P* < .01. ^c^*P* < .001.

Bivariate correlations between depressive symptoms and disturbed sleep are reported in eTable 6 in the Supplement. Figure 1 shows the standardized path coefficients (β) and the fit indices of the cross-lagged model. The stability path estimates for disturbed sleep (range β = 0.42 [95% CI, 0.35-0.48] to β = 0.47 [95% CI, 0.42-0.52]) and depressive symptoms (range β = 0.36 [95% CI, 0.29-0.42] to β = 0.57 [95% CI, 0.53-0.61]) were fairly stable across time. The correlations between disturbed sleep and depressive symptoms were significant at each time point (*r* = 0.18 at 5 years, *r* = 0.09 at 7 years, and *r* = 0.16 at 8 years). Cross-lagged path estimates showed that depressive symptoms were significantly associated with later disturbed sleep, and disturbed sleep was associated with later depressive symptoms at all time points (range β = 0.07 [95% CI 0.02-0.12] to β = 0.15 [95% CI, 0.09-0.19]), suggesting a developmental cascade throughout childhood. Also, there were significant associations between depressive symptoms at 5 and 8 years (*b* = 0.27; SE = 0.038; β = 0.25 [95% CI, 0.19-0.32]; *P* < .001), and between disturbed sleep at 5 and 8 years (*b* = 0.23; SE = 0.031; β = 0.22 [95% CI, 0.17-0.29]; *P* < .001). Among covariates, only paths between maternal depression and both disturbed sleep and depressive symptoms were significant (β = 0.11 [95% CI, 0.06-0.17]; *P* < .001; and β = 0.22 [95% CI, 0.16-0.27]; *P* < .001).

### Adolescence

**Figure 2.**
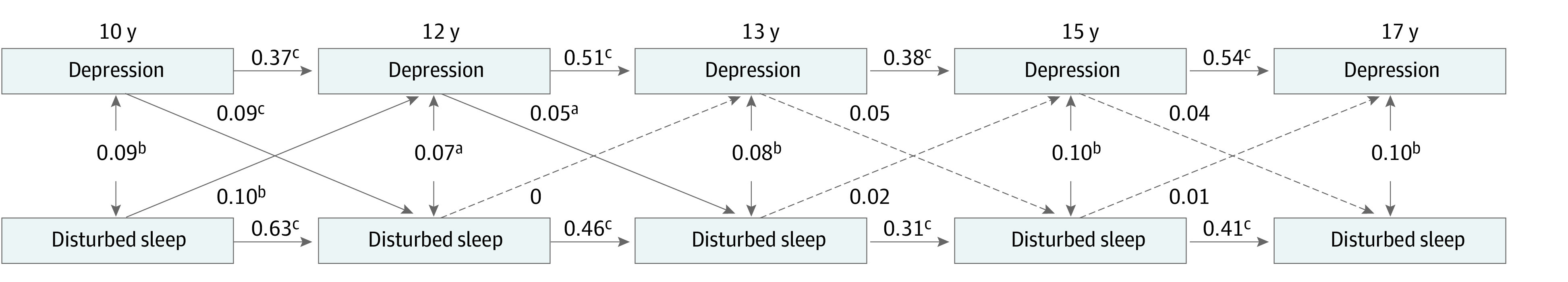
Estimates From the Cross-Lagged Model for the Reciprocal Association Between Disturbed Sleep and Depression Across Adolescence All estimates are standardized. Dashed arrows indicate nonsignificant paths. Fit statistics: χ^2^_28_ = 48.842; *P* = .009; root mean square error of approximation = 0.026 (95% CI, 0.013-0.038); comparative fit index = 0.993; Tucker Lewis index = 0.985. Covariates were socioeconomic status and maternal depression. *P* < .05 was considered statistically significant. Data are courtesy of the Quebec Institute of Statistics. ^a^*P* < .05. ^b^*P* < .01. ^c^*P* < .001.

In the adolescent analysis, 1113 individuals were included (595 [53.5%] female) (Table 1). Participants were from a higher SES and were more often girls compared with the nonparticipants (1007 participants; *t*_2115_ = −7.11; χ^2^_1_ = 18.17; *P* < .001). Descriptive statistics across time points for the study variables are shown in Table 3. Bivariate correlations between depressive symptoms and disturbed sleep are reported in eTable 7 in the Supplement. Figure 2 shows the β coefficients and the fit indices of the cross-lagged model. The stability path estimates for disturbed sleep (range β = 0.31 [95% CI, 0.23 to 0.39] to β = 0.63 [95% CI, 0.58 to 0.67]) and depressive symptoms (β = 0.37 [95% CI, 0.32 to 0.42] to β = 0.54 [95% CI, 0.49 t 0.60]) at baseline were fairly stable across all 5 time points. The correlation between depressive symptoms and disturbed sleep was significant at each time point (*r* range, 0.07 to 0.10). Cross-lagged path estimates were significant from depressive symptoms to disturbed sleep (β = 0.09 [95% CI, 0.04 to 0.14]; *P* < .001) and vice versa (β = 0.10 [95% CI, 0.04 to 0.16]; *P* = .001) between 10 and 12 years. Between 12 and 13 years, depressive symptoms were modestly associated with disturbed sleep (β = 0.05 [95% CI, 0.001 to 0.10], *P* = .04) but the reverse association was not significant. Cross-lagged estimates were nonsignificant after 13 years. Among covariates, significant paths were found between maternal depression and both disturbed sleep and depressive symptoms (β = 0.17 [95% CI, 0.11 to 0.24]; *P* < .001 and β = 0.08 [95% CI, 0.01 to 0.15]; *P* = .02, respectively), and between SES and depressive symptoms (β = −0.15 [95% CI, −0.21 to −0.09]; *P* < .001). Compared with the unconstrained model, the fit of either the sex-invariant or the puberty-by-sex-invariant model was not deteriorated (difference between 2 nested models, χ^2^_33_ = 29.468; *P* = .64 and χ^2^_99_ = 99.781; *P* = .46, respectively), indicating that the associations between depressive symptoms and disturbed sleep did not vary as a funciton of either sex or puberty-by-sex. Cross-lagged paths were identical when listwise deletion was used as an alternative missing data strategy, in both childhood and adolescence.

**Table 3.** Descriptive Statistics in the Adolescence Sample^a^

Time point and variable	Adolescents, No. (N = 1113)	Total score, mean (SD) [range]
10 y		
Disturbed sleep	941	1.87 (0.76) [0.00 to 10.00]
Depression	1024	2.81 (1.61) [0.00 to 10.00]
Maternal depression	951	1.33 (1.28) [0.00 to 10.00]
Socioeconomic status	1113	0.09 (0.91) [−2.59 to 2.78]
12 y		
Disturbed sleep	1044	1.76 (0.74) [0.00 to 10.00]
Depression	1080	2.33 (1.68) [0.00 to 10.00]
13 y		
Disturbed sleep	950	1.76 (0.72) [0.00 to 10.00]
Depression	1047	2.22 (1.82) [0.00 to 10.00]
15 y		
Disturbed sleep	998	1.50 (0.69) [0.00 to 10.00]
Depression	1082	3.28 (2.18) [0.00 to 10.00]
17 y		
Disturbed sleep	894	1.42 (0.74) [0.00 to 10.00]
Depression	976	3.67 (2.26) [0.00 to 10.00]

^a^ Data are courtesy of the Quebec Institute of Statistics.

Cross-lagged paths using the insomnia index are reported in eResults 2, eFigure 1, and eFigure 2 in the Supplement. Results from the cross-lagged models (1) 5, 7, 8, and 10 years; (2) 12, 13, 15, and 17 years; and (3) 5, 7, 8, 10, 12, 13, 15, and 17 years are reported in eResults 3, eFigure 3, eFigure 4, and eFigure 5 in the Supplement.

## Discussion

To our knowledge, this cohort study is the first to report developmental cascades between disturbed sleep and depressive symptoms throughout childhood and early adolescence. Our finding suggests that disturbed sleep makes a significant contribution to the stability over time of depressive symptoms across childhood and early adolescence, which, in turn, is associated with ongoing disturbed sleep.

To date, 2 studies^50,51^ that we know of have tested cross-lagged associations over 3 or more time points in childhood and developmental cascades were not reported. Quach et al^50^ found that disturbed sleep is consistently associated with increased depression from preschool to 12 years, and bidirectionality was found only from 6 to 7 years. Although the study sample and measurements were similar to ours, analyses were not controlled for any covariates, which could plausibly account for the discrepancy of the results. Foley et al^51^ found no bidirectional effects but only a unidirectional association from disturbed sleep to depression between preschool and grade 1. Differently from our study, the authors tested the reciprocal effects of disturbed sleep, depression, and social competence, therefore adding 1 construct in the cross-lagged model, which could partly explain the discrepancy of the results. In adolescence, we found no developmental cascades, which was in line with 3^31,37,49^ previous studies but differed from a fourth study,^48^ and a bidirectional association between 10 and 12 years in line with 1 previous study.^37^ Starting at 13 years, cross-lagged associations were not significant at any time points. Finally, effects held across sexes in line with previous results^31,37,49,51^ and were unaffected by including pubertal status as a moderator in the model.

Taken together, our findings suggest that there might be a sensitive window between 5 and 12 years for sleep interventions to mitigate the emergence and/or growth of later depressive symptoms. Up to now, randomized clinical trials of sleep interventions in depressed adolescents have targeted mid adolescence (ie, 15 years)^42,71^ and yielded small effect sizes (pooled standardized mean difference = −0.27 [95% CI, −0.50 to −0.04]).^42^ Starting prevention efforts in childhood may have a similar, or even greater impact on depression compared with treatment initiated in adolescence,^42,71^ because it would reduce the risk before depression consolidates. To date, we are not aware of any randomized clinical trials of sleep interventions specifically addressing depressed children. To expand our understanding, randomized clinical trials with long-term follow-up and in children with different levels of baseline depression are needed. According to our findings, a sample of 100 individuals would be adequately powered for a sleep intervention for the prevention of depression in a children community sample.

The magnitude of the cross-lagged estimates that we found was overall small (β range, 0.05-0.15), but quite in line with previous reports^39^ and with etiological models of depression. Indeed, multiple risk factors, each with a small effect, underlie the vulnerability to depression,^72^ and this may partly explain why sleep interventions, albeit having a significant effect, are limited in preventing depression in adolescents.^42,71^ The potential for a larger impact could be achieved by simultaneously addressing multiple risk factors of depression. Disturbed sleep often co-occurs with other lifestyle risk behaviors associated with depression, including poor diet,^73^ poor physical activity,^74^ and excessive recreational screen time.^75^ Future research should examine whether a multiple health behavior change approach could be a viable option for depression prevention.^76^

Understanding the biological mechanisms underlying the developmental cascade linking disturbed sleep and depression is of paramount importance. Key outstanding questions include whether an early sensitive window of vulnerability can be identified, which could explain why cascade processes concentrate in early childhood and fade in adolescence. Both conditions^77^ are heritable^4^ and share common genetic risk factors.^78^ Both disturbed sleep^79^ and depression^80^ have been associated bidirectionally with inflammatory processes, suggesting that inflammation may be one mechanism driving the spiraling process. Interestingly, treating disturbed sleep and depression attenuates inflammation,^81,82^ and using anti-inflammatory medications to treat depression seems promising in adults.^83^ Future research is needed to determine whether patterns of proinflammatory responses may be one of the biological mechanisms through which disturbed sleep accelerates depression and whether it can be targeted for depression prevention in children and adolescents.

### Limitations

There are some limitations in this study. First, participants were from a higher SES and more often girls than nonparticipants, which potentially limits the generalizability of our results. Second, the questionnaire for depression did not rate symptoms’ severity. Furthermore, depressive symptoms were parent-reported in childhood and self-reported in adolescence, which may have accounted for the differences found between childhood and adolescent findings. However, the high stability of scores suggests that both adolescent and parent-report measures tapped into the same constructs. Third, in childhood, parents reported on both disturbed sleep and depressive symptoms, which might have implied perception bias. Fourth, the questionnaire for disturbed sleep was not validated. Furthermore, it was parent-reported, which have been shown to be less accurate compared with self-report and objective measurements of sleep.^84^ Results may need to be replicated with objective measurements and/or validated questionnaires. However, self-reports remain the measure of choice in community surveys. Additionally, for disturbed sleep during childhood, there was a slight difference in the pattern of missing data related to time since enrollment. Although the analytical strategy that was used to deal with missing data mitigates the effects of attrition, we cannot exclude that missing data may have affected the results in the childhood analysis.

## Conclusions

In conclusion, bidirectional and developmental cascades linking disturbed sleep and depressive symptoms in childhood and early adolescence emphasize the importance of identifying and treating incipient disturbed sleep as early as possible to prevent spiraling effects on depression. More randomized clinical trials testing interventions possibly targeting common pathways are needed to bolster depression prevention. Because of the reliance on self-report data, results should be interpreted with caution.

## Notes

Supplement. **eMethods 1.** Characteristic of the Quebec Longitudinal Study of Child Development (QLSCD) **eMethods 2.** Seven-Item Parent-Reported Questionnaire to Assess Disturbed Sleep in Children and Adolescents **eMethods 3.** Eight-Item Parent-Reported Questionnaire to Assess Depression in Children **eMethods 4.** Ten-Item Self-Reported Questionnaire to Assess Depression in Adolescents **eMethods 5.** Measurements’ Details for Covariates/Moderators, i.e. Socioeconomic Status (SES), Maternal Depression and Pubertal Status **eResults 1.** Missing Data Pattern for Outcome Variables in Childhood and Adolescence **eResults 2.** Cross-Lagged Paths Between the Insomnia Index and Depressive Symptoms in Childhood and Adolescence **eResults 3.** Cross-Lagged Paths Between the Disturbed Sleep and Depressive Symptoms Including the Following Time-Points: (1) 5, 7, 8, 10 years, (2) 12, 13, 15 and 17 years, and (3) 5, 7, 8, 10, 12, 13, 15 and 17 Years **eTable 1.** Tanner Stage Distribution for Adrenarche and Gonadarche for Males and Females **eTable 2.** Rates of Missingness for Depression and Disturbed Sleep in Childhood **eTable 3.** Characteristics of the Study Variables at Each Time Point in the Complete Versus With-Missing-Data Subgroups in the Childhood Sample **eTable 4.** Rates of Missingness for Depression and Disturbed Sleep in Adolescence **eTable 5.** Characteristics of the Study Variables at Each Time Point in the Complete Versus With-Missing-Data Subgroups in the Adolescence Sample **eTable 6.** Correlation Matrix Between Disturbed Sleep and Depression in Childhood **eTable 7.** Correlation Matrix Between Disturbed Sleep and Depression in the Adolescence Sample **eFigure 1.** Estimates From the Cross-Lagged Model for the Reciprocal Associations Between the Insomnia Index and Depression Across Childhood (n=1689) **eFigure 2.** Estimates From the Cross-Lagged Model for the Reciprocal Association Between the Insomnia Index and Depression Across Adolescence (n=1113) **eFigure 3.** Estimates From the Cross-Lagged Model for the Reciprocal Associations Between Disturbed Sleep and Depression Across Childhood Including Time-Points 5, 7, 8 and 10 Years (n=1689) **eFigure 4.** Estimates From the Cross-Lagged Model for the Reciprocal Association Between Disturbed Sleep and Depression Across Adolescence Including Time-Points 12, 13, 15 and 17 Years (n=1113) **eFigure 5.** Estimates From the Cross-Lagged Model for the Reciprocal Associations Between Disturbed Sleep and Depression From 5 to 17 Years of Age (n=1085)
