# Supplementary material for: Testing Bidirectional, Longitudinal Associations Between Disturbed Sleep and Depressive Symptoms in Children and Adolescents Using Cross-Lagged Models
Source: JAMA Netw Open. 2022 Aug 22;5(8):e2227119. doi: 10.1001/jamanetworkopen.2022.27119 (PMC9396361; doi:10.1001/jamanetworkopen.2022.27119)
Supplement: Supplement. — eMethods 1. Characteristic of the Quebec Longitudinal Study of Child Development (QLSCD) eMethods 2. Seven-Item Parent-Reported Questionnaire to Assess Disturbed Sleep in Children and Adolescents eMethods 3. Eight-Item Parent-Reported Questionnaire to Assess Depression in Children eMethods 4. Ten-Item Self-Reported Questionnaire to Assess Depression in Adolescents eMethods 5. Measurements’ Details for Covariates/Moderators, i.e. Socioeconomic Status (SES), Maternal Depression and Pubertal Status eResults 1. Missing Data Pattern for Outcome Variables in Childhood and Adolescence eResults 2. Cross-Lagged Paths Between the Insomnia Index and Depressive Symptoms in Childhood and Adolescence eResults 3. Cross-Lagged Paths Between the Disturbed Sleep and Depressive Symptoms Including the Following Time-Points: (1) 5, 7, 8, 10 years, (2) 12, 13, 15 and 17 years, and (3) 5, 7, 8, 10, 12, 13, 15 and 17 Years eTable 1. Tanner Stage Distribution for Adrenarche and Gonadarche for Males and Females eTable 2. Rates of Missingness for Depression and Disturbed Sleep in Childhood eTable 3. Characteristics of the Study Variables at Each Time Point in the Complete Versus With-Missing-Data Subgroups in the Childhood Sample eTable 4. Rates of Missingness for Depression and Disturbed Sleep in Adolescence eTable 5. Characteristics of the Study Variables at Each Time Point in the Complete Versus With-Missing-Data Subgroups in the Adolescence Sample eTable 6. Correlation Matrix Between Disturbed Sleep and Depression in Childhood eTable 7. Correlation Matrix Between Disturbed Sleep and Depression in the Adolescence Sample eFigure 1. Estimates From the Cross-Lagged Model for the Reciprocal Associations Between the Insomnia Index and Depression Across Childhood (n=1689) eFigure 2. Estimates From the Cross-Lagged Model for the Reciprocal Association Between the Insomnia Index and Depression Across Adolescence (n=1113) eFigure 3. Estimates From the Cross-Lagged Model for the Reciprocal Associati [file jamanetwopen-e2227119-s001.pdf]

Supplemental Online Content

Marino C, Andrade B, Montplaisir J, et al. Testing bidirectional, longitudinal associations between disturbed sleep and depressive symptoms in children and adolescents using cross-lagged models. *JAMA Netw Open*. 2022;5(8):e2227119. doi:10.1001/jamanetworkopen.2022.27119

eMethods 1. Characteristic of the Quebec Longitudinal Study of Child Development (QLSCD)
eMethods 2. Seven-Item Parent-Reported Questionnaire to Assess Disturbed Sleep in Children and Adolescents
eMethods 3. Eight-Item Parent-Reported Questionnaire to Assess Depression in Children
eMethods 4. Ten-Item Self-Reported Questionnaire to Assess Depression in Adolescents
eMethods 5. Measurements' Details for Covariates/Moderators, i.e. Socioeconomic Status (SES), Maternal Depression and Pubertal Status
eResults 1. Missing Data Pattern for Outcome Variables in Childhood and Adolescence
eResults 2. Cross-Lagged Paths Between the Insomnia Index and Depressive Symptoms in Childhood and Adolescence
eResults 3. Cross-Lagged Paths Between the Disturbed Sleep and Depressive Symptoms Including the Following Time-Points: (1) 5, 7, 8, 10 years, (2) 12, 13, 15 and 17 years, and (3) 5, 7, 8, 10, 12, 13, 15 and 17 Years
eTable 1. Tanner Stage Distribution for Adrenarche and Gonadarche for Males and Females
eTable 2. Rates of Missingness for Depression and Disturbed Sleep in Childhood
eTable 3. Characteristics of the Study Variables at Each Time Point in the Complete Versus With-Missing-Data Subgroups in the Childhood Sample
eTable 4. Rates of Missingness for Depression and Disturbed Sleep in Adolescence
eTable 5. Characteristics of the Study Variables at Each Time Point in the Complete Versus With-Missing-Data Subgroups in the Adolescence Sample
eTable 6. Correlation Matrix Between Disturbed Sleep and Depression in Childhood
eTable 7. Correlation Matrix Between Disturbed Sleep and Depression in the Adolescence Sample
eFigure 1. Estimates From the Cross-Lagged Model for the Reciprocal Associations Between the Insomnia Index and Depression Across Childhood (n=1689)
eFigure 2. Estimates From the Cross-Lagged Model for the Reciprocal Association Between the Insomnia Index and Depression Across Adolescence (n=1113)
eFigure 3. Estimates From the Cross-Lagged Model for the Reciprocal Associations Between Disturbed Sleep and Depression Across Childhood Including Time-Points 5, 7, 8 and 10 Years (n=1689)

© 2022 Marino C et al. *JAMA Network Open.*

eFigure 4. Estimates From the Cross-Lagged Model for the Reciprocal Association Between Disturbed Sleep and Depression Across Adolescence Including Time-Points 12, 13, 15 and 17 Years (n=1113)
eFigure 5. Estimates From the Cross-Lagged Model for the Reciprocal Associations Between Disturbed Sleep and Depression From 5 to 17 Years of Age (n=1085)

This supplemental material has been provided by the authors to give readers additional information about their work.

eMethods 1

Characteristic of the Quebec Longitudinal Study of Child Development (QLSCD)

The Quebec Longitudinal Study of Child Development (QLSCD) is a population-based representative sample of children in the Canadian Province of Quebec, born between October 1997 and July 1998. The cohort study was designed to investigate the early childhood factors on later developmental outcomes.

The cohort recruited singletons born between October 1997 and July 1998 in each administrative region of the Canadian province of Quebec, except those born in Northern Quebec, Cree Territory, Inuit Territory, and Native reserves (2.2% of all births).

Infants were excluded if born with severe medical illnesses, born <24 or >42 weeks of gestation, from families living in Aboriginal territories or remote regions of Québec, or from non-English-and non-French-speaking families. Of 2940 families initially recruited, 2223 families participated in the study when they were 5 months old and 2120 agreed to participate in the longitudinal follow-up. Participants are being evaluated almost annually and assessments are currently taking place at age 21 years.

Data were collected in four phases, of which Phase 2 (2003–2010, ages 6, 7, 8, 10, and 12 years) and Phase 3 (2011–2015, ages 13, 15, and 17 years) were the ones inherent to our study.

eMethods 2

Seven-item parent-reported questionnaire to assess disturbed sleep in children and adolescents

1. Indicate how long in total he/she sleeps during the NIGHT (on average). Do not count the hours that your child is awake
2. Indicate how long in total he/she is awake during the NIGHT (on average).
3. In general, how many hours does your child sleep during the DAY (total of all naps)?
4. Does your child TALK in his/her sleep?
5. Does your child WALK in his/her sleep?
6. Does your child have NIGHTMARES?
7. Does your child have NIGHTTERRORS (wakes up suddenly, crying, sometimes drenched in sweat and confused)?

Each item was rated on a 4-point scale (1=never, 2=sometimes, 3=often, 4=always), except for sleep duration and time awake in bed, which were reported in number of hours and minutes, and were re-scaled to vary between 1 and 4. Sleep duration was reverse-coded to have the same direction of the other items and in the assumption that shorter sleep duration is a risk factor for depression [49].

eMethods 3

Eight-item parent-reported questionnaire to assess depression in children

1. Seemed to be unhappy or sad?
2. Was not as happy as other children?
3. Was unable to concentrate, could not pay attention for long?
4. Was too fearful or anxious?
5. Was worried?
6. Cried a lot?
7. Was nervous, highstrung or tense?
8. Had trouble enjoying HIM/HER self?

Each item was rated on a 3-point scale, (1=never, 2=sometimes, 3=often). Cronbach's alphas at 5, 7 and 8 years were 0.70, 0.72 and 0.73, respectively. A mean score was computed, and the score re-scaled to range 0-10. The higher the score, the more depressive symptoms.

© 2022 Marino C et al. *JAMA Network Open.*

eMethods 4

Ten-item self-reported questionnaire to assess depression in adolescents

1. I am unhappy or sad
2. I am not as happy as other people of my age
3. I can't concentrate, I can't pay attention
4. I am too fearful or nervous
5. I worry a lot
6. I cry a lot
7. I am nervous, high strung or tense
8. I have trouble enjoying myself
9. I have temper tantrums or easily get angry
10. I am not interested in doing activities with other children

Each item was rated on a 3-point scale (1=never, 2=sometimes, 3=often). Cronbach's alphas at 10, 12, 13, 15 and 17 years were 0.74, 0.78, 0.82, 0.88 and 0.89, respectively.

eMethods 5

Measurements' details for covariates/moderators, i.e. socio-economic stauts (SES), maternal depression and pubertal status

SES was the standardized composite of 5 items enquiring about parental education, occupational status, and household income (range, −3 to 3, centered at 0, higher scores indicating higher SES) [63].

For maternal depression, mothers reported how often they experienced depressive symptoms on a 4-point scale (0=never, 1=sometimes, 2=often, 3=always) using a shortened version of the Center for Epidemiologic Studies Depression Scale 64. Scores ranged from 0 to 10, with higher scores indicating higher depression.

For pubertal status, 12 and 13-year-old adolescents were provided with sex-appropriate drawings of secondary sex characteristics based on Tanner stages of pubertal development. Adolescents were asked to choose which of the drawings were most like them 65, and Tanner scores were assigned from I, prepubertal, to V, full pubertal, accordingly (eTable 1 in the Supplement). Self-rated pubertal status has been shown to be sufficiently accurate for large epidemiologic studies 66. The average scores were used as an overall index of pubertal status at each time point (12 years: M=2.76, SD=0.85; 13 years: M=3.50, SD=0.82). Given the high correlation of pubertal status between ages 12 and 13 years (r=0.72, p<0.001), the average score was used in further analyses (M=3.08, SD=0.81).

© 2022 Marino C et al. *JAMA Network Open.*

eResults 1: Missing data pattern for outcome variables in childhood and adolescence.

Missing data pattern for outcome variables in childhood.
Rate of missingness for depressive symptoms and disturbed sleep at each time point are provided in eTable 2 in the Supplement.
eTable 3 in the Supplement reports the characteristics of the study variables at each time point in the complete versus with-missing data subgroups. At 6 years of age, children with complete data were significantly less depressed compared to children with missing data. Furthermore, SES was lower and maternal depression was higher in those with missing data compared to those with complete data.
The missing data pattern across the 3 time points was MCAR for depressive symptoms ($\chi 2$ = 14.90, df = 9, p = .09) and missing at random (MAR) for sleep ($\chi 2$ = 19.18, df = 9, p = .02). As for depressive symptoms, there was not systematic differences in missing data related to time since enrollment. As for disturbed sleep, there was only a slight difference in missing data related to time since enrollment

Missing data pattern for outcome variables in adolescence.
Rates of missingness for depressive symptoms and disturbed sleep at each time point are provided in eTable 5 in the Supplement.
eTable 6 in the Supplement reports the characteristics of the study variables at each time point in the complete versus with-missing data subgroups. At 10 years of age, adolescents with complete data were significantly less depressed compared to adolescents with missing data. At 15 years of age, adolescents with complete data had significantly less disturbed sleep compared to adolescents with missing data. Furthermore, SES was lower and maternal depression was higher in those with missing data compared to those with complete data.
The missing data pattern across the 5 time points was MCAR for depressive symptoms ($\chi 2$ = 42.45, df = 50, p =.77) and for sleep ($\chi 2$ = 56.84, df = 50, p =.24). As for disturbed sleep and depressive symptoms, there were not systematic differences in missing data related to time since enrollment.

eResults 2: Cross-lagged paths between the insomnia index and depressive symptoms in childhood and adolescence

Cross-lagged paths between the insomnia index and depressive symptoms in childhood.

Findings showed that depressive symptoms were significantly associated with later insomnia at each time point, whereas insomnia was not associated with later depressive symptoms at any time points (eFigure 1 in the Supplement).

Cross-lagged paths between the insomnia index and depressive symptoms in adolescence.

Findings showed two unidirectional longitudinal associations, respectively from insomnia at 10 years to depressive symptoms at 12 years, and from depressive symptoms at 12 years to insomnia at 13 years (eFigure 3 in the Supplement).

eResults 3: Cross-lagged paths between disturbed sleep and depressive symptoms including the following time-points i.e. (1) 5, 7, 8, 10 years, (2) 12, 13, 15 and 17 years, and (3) 5, 7, 8, 10, 12, 13, 15 and 17 years.

Cross-lagged paths between disturbed sleep and depressive symptoms in childhood including time points 5, 7, 8 and 10 years.

© 2022 Marino C et al. *JAMA Network Open.*

Findings showed that depressive symptoms were significantly associated with later disturbed sleep, and disturbed sleep was associated with later depressive symptoms between 5 and 8 years (eFigure 2 in the Supplement).

Cross-lagged paths between disturbed sleep and depressive symptoms in adolescence including time-points 12, 13, 15 and 17 years.

Findings showed a significant path between depressive symptoms at 12 years and disturbed sleep at 13 years (eFigure 4).

Cross-lagged paths between disturbed sleep and depressive symptoms including all time-points, i.e. 5, 7, 8, 10, 12, 13, 15 and 17 years.

Findings showed longitudinal, reciprocal associations between depressive symptoms and disturbed sleep between 5 and 8 years, and unidirectional associations between depressive symptoms at 8 years and disturbed sleep at 10 years, and between depressive symptoms at 10 years and disturbed sleep at 12 years (eFigure 5 in the Supplement).

© 2022 Marino C et al. *JAMA Network Open.*

eTable 1. Tanner stage distribution for adrenarche and gonadarche for males and females

	12 years				13 years			
	**Adrenarche, males (n=642)**	**Adrenarche, females (n=699)**	**Gonadarche, males (n=641)**	**Gonadarche, females (n=699)**	**Adrenarche, males (n=538)**	**Adrenarche, females (n=651)**	**Gonadarche, males (n=637)**	**Gonadarche, females (n=655)**
**Tanner I**	79	52	45	40	5	15	9	10
**Tanner II**	261	157	278	151	91	38	94	36
**Tanner III**	211	260	219	343	203	164	197	261
**Tanner IV**	81	203	89	159	195	303	197	299
**Tanner V**	10	27	10	6	44	131	40	49

© 2022 Marino C et al. *JAMA Network Open.*

eTable 2: Rates of missingness for depression and disturbed sleep at each time point in childhood

Study variable	% missing
**Depression, 5 years**	3.37
**Depression, 6 years**	15.45
**Depression, 8 years**	17.58
**Disturbed sleep, 5 years**	14.86
**Disturbed sleep, 6 years**	22.62
**Disturbed sleep, 8 years**	24.46

© 2022 Marino C et al. *JAMA Network Open.*

eTable 3: Characteristics of the study variables at each time point in the complete versus with-missing data subgroups in the childhood sample

Study variable	Complete data	With-missing data	p-value
	N=971(57.5%)	N=718(42.5%)	
	M (SD)	*M (SD)*	
**Depression, 5 years**	2.33 (1.49)	2.32 (1.44)	0.93
**Depression, 6 years**	2.37 (1.52)	2.59 (1.56)	0.01
**Depression, 8 years**	2.49 (1.59)	2.58 (1.54)	0.31
**Disturbed Sleep, 5 years**	1.50 (0.20)	1.49 (0.21)	0.54
**Disturbed Sleep, 6 years**	1.46 (0.19)	1.47 (0.21)	0.90
**Disturbed Sleep, 8 years**	1.38 (0.20)	1.40 (0.20)	0.13
**Sex (male%)**	52.32	47.91%	0.07
**Socio-economic status**	0.13 (0.91)	-0.09 (0.99)	<0.001
**Maternal depression**	1.42 (1.61)	1.66 (1.82)	0.02

Differences between groups were tested by T-test, 2 sided for all variables except for Sex, for which a chi-squared test was used

© 2022 Marino C et al. *JAMA Network Open.*

eTable 4: Rates of missingness for depression and disturbed sleep at each time point in adolescence

10 years	% missing
**Depression, 10 years**	7.99
**Depression, 12 years**	3.23
**Depression, 13 years**	6.20
**Depression, 15 years**	3.05
**Depression, 17 years**	12.58
**Disturbed sleep, 10 years**	15.72
**Disturbed sleep, 12 years**	6.47
**Disturbed sleep, 13 years**	14.91
**Disturbed sleep, 15 years**	10.60
**Disturbed sleep, 17 years**	19.94

© 2022 Marino C et al. *JAMA Network Open.*

eTable 5: Characteristics of the study variables at each time point in the complete versus with-missing data subgroups in the adolescence sample

Study variable	Complete data	With-missing data	p-value
	N=533(47.9%)	N=580(52.1%)	
	M (SD)	*M (SD)*	
**Depression, 10 years**	2.69 (1.57)	2.95 (1.63)	<0.01
**Depression, 12 years**	2.29 (1.65)	2.38 (1.70)	0.39
**Depression, 13 years**	2.16 (1.77)	2.29 (1.87)	0.27
**Depression, 15 years**	3.22 (2.14)	3.34 (2.22)	0.39
**Depression, 17 years**	3.59 (2.23)	3.75 (2.30)	0.26
**Disturbed sleep, 10 years**	1.55 (0.22)	1.57 (0.24)	0.13
**Disturbed sleep, 12 years**	1.52 (0.21)	1.54 (0.24)	0.12
**Disturbed sleep, 13 years**	1.52 (0.21)	1.54 (0.22)	0.38
**Disturbed sleep, 15 years**	1.44 (0.19)	1.47 (0.22)	0.03
**Disturbed sleep, 17 years**	1.43 (0.22)	1.43 (0.22)	0.90
**Sex**[a]	54.03	51.21%	0.12
**Socio-economic status**	0.26 (0.87	-0.06 (0.91)	0.001
**Maternal depression**	1.21 (1.19)	1.48 (1.36)	<0.001

Differences between groups were tested by T-test, 2 sided for all variables except Sex, for which a chi-squared test was used

© 2022 Marino C et al. *JAMA Network Open.*

eTable 6. Correlation matrix between disturbed sleep and depression in childhood.

	Depression, 5 years	Depression, 7 years	Depression, 8 years	Disturbed sleep, 5 years	Disturbed sleep, 7 years	Disturbed sleep, 8 years
**Depression, 5 years**	-					
**Depression, 7 years**	0.59 **	-				
**Depression, 8 years**	0.48 **	0.52 **	-			
**Disturbed sleep, 5 years**	0.20 **	0.21 **	0.18 **	-		
**Disturbed sleep, 7 years**	0.23 **	0.24 **	0.21 **	0.49 **	-	
**Disturbed sleep, 8 years**	0.18 **	0.21 **	0.27 **	0.43 **	0.54 **	-

*p<0.05; **p<0.01; ***p<0.001. Data are courtesy of the Quebec Institute of Statistics.

© 2022 Marino C et al. *JAMA Network Open.*

eTable 7. Correlation matrix between disturbed sleep and depression in the adolescence sample.

	Depression, 10 years	Depression, 12 years	Depression, 13 years	Depression, 15 years	Depression, 17 years	Disturbed sleep, 10 years	Disturbed sleep, 12 years	Disturbed sleep, 13 years	Disturbed sleep, 15 years	Disturbed sleep, 17 years
**Depression, 10 years**	-									
**Depression, 12 years**	0.38 **	-								
**Depression, 13 years**	0.32 **	0.56 **	-							
**Depression, 15 years**	0.20 **	0.37 **	0.48 **	-						
**Depression, 17 years**	0.16 **	0.34 **	0.41 **	0.62 **	-					
**Disturbed sleep, 10 years**	0.09 **	0.13 **	0.12 **	0.10 **	0.07 *	-				
**Disturbed sleep, 12 years**	0.15 **	0.17 **	0.10 **	0.12 **	0.11 **	0.63 **	-			
**Disturbed sleep, 13 years**	0.07 *	0.17 **	0.15 **	0.12 **	0.13 **	0.59 **	0.64 **	-		
**Disturbed sleep, 15 years**	0.13 **	0.16 **	0.15 **	0.16 **	0.13 **	0.53 **	0.58 **	0.57 **	-	
**Disturbed sleep, 17 years**	0.10 **	0.18 **	0.19 **	0.14 **	0.18 **	0.47 **	0.50 **	0.46 **	0.60 **	

*p<0.05; **p<0.01; ***p<0.001. Data are courtesy of the Quebec Institute of Statistics.

© 2022 Marino C et al. *JAMA Network Open.*

eFigure 1. Estimates from the cross-lagged model for the reciprocal associations between the insomnia index and depression across childhood (n=1689).

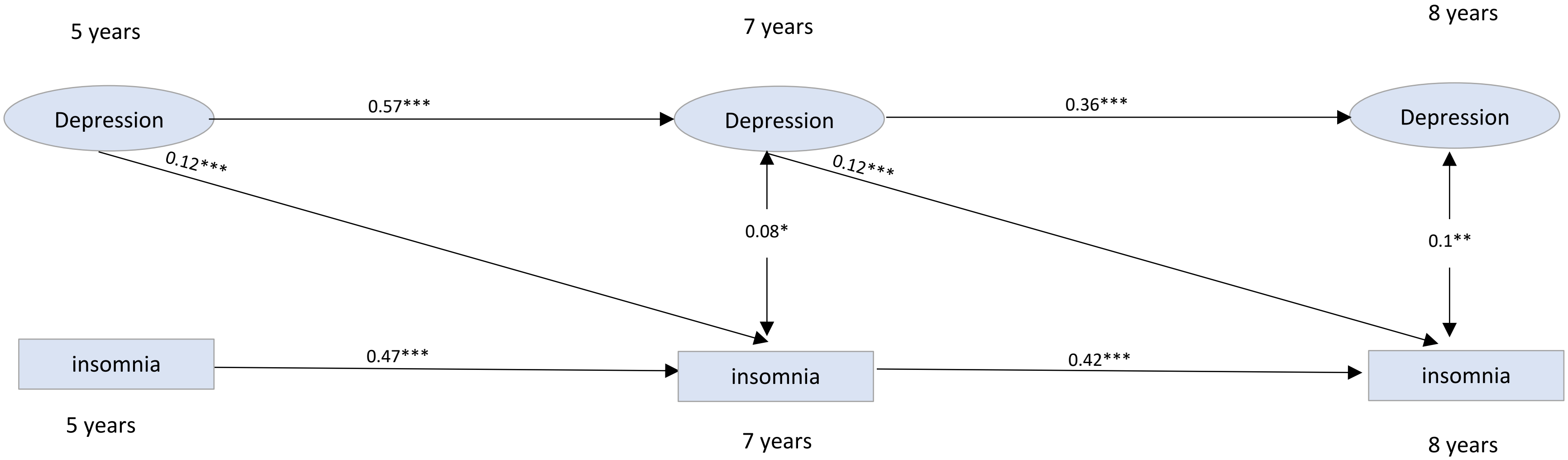

© 2022 Marino C et al. *JAMA Network Open.*

eFigure 1 legend

Source. Data are courtesy of the Quebec Institute of Statistics.
All estimates are standardized. Fit statistics: Chi-square=66.678(16), $p<.001$; RMSEA=0.043[0.033, 0.054]; CFI=0.961; TLI=0.919. CFI = comparative fit index; TLI = Tucker Lewis index; RMSEA= root mean square error of approximation. Covariates were socio-economic status and maternal depression. Non-significant paths are not shown.
P-values less than 0.05 were considered statistically significant. *$p<0.05$; **$p<0.01$; ***$p<0.001$.

© 2022 Marino C et al. *JAMA Network Open.*

eFigure 2. Estimates from the cross-lagged model for the reciprocal association between the insomnia index and depression across adolescence (n=1113).

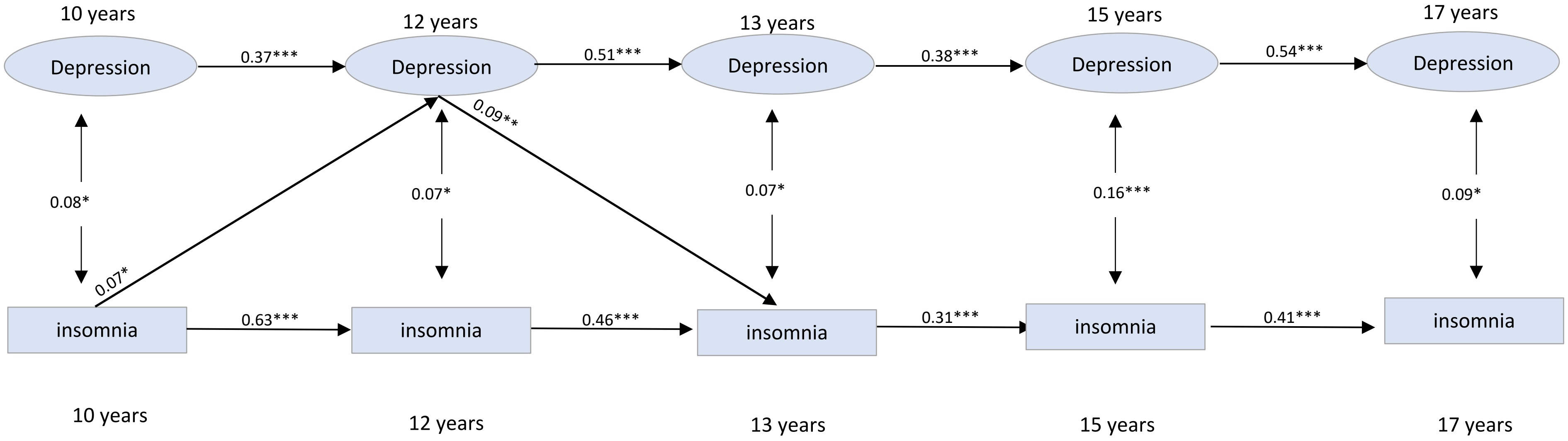

© 2022 Marino C et al. *JAMA Network Open.*

eFigure 2 legend

Source. Data are courtesy of the Quebec Institute of Statistics.
All estimates are standardized. Fit statistics: Chi-square=53.710(28), p=0.0024; RMSEA=0.029[0.017, 0.040]; CFI=0.988; TLI=0.972. CFI = comparative fit index; TLI = Tucker Lewis index; RMSEA= root mean square error of approximation. Covariates were socio-economic status and maternal depression. Non-significant paths are not shown.
P-values less than 0.05 were considered statistically significant. *p<0.05; **p<0.01; ***p<0.001.

© 2022 Marino C et al. *JAMA Network Open.*

eFigure 3. Estimates from the cross-lagged model for the reciprocal associations between disturbed sleep and depression across childhood including 10 years of age (n=1689) .

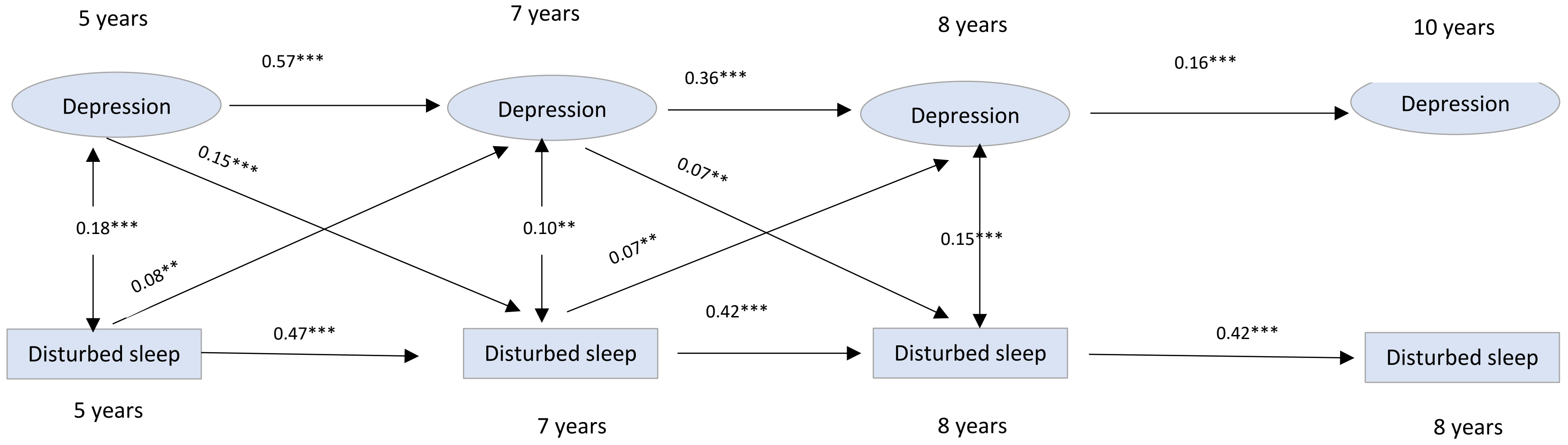

© 2022 Marino C et al. *JAMA Network Open.*

eFigure 3 legend

Source. Data are courtesy of the Quebec Institute of Statistics.
All estimates are standardized. Fit statistics: Chi-square=88.138(28), p<.001; RMSEA=0.036 [0.027, 0.044]; CFI=0.976; TLI=0.955. CFI = comparative fit index; TLI = Tucker Lewis index; RMSEA= root mean square error of approximation. Covariates were socio-economic status and maternal depression. Non-significant paths are not shown.
P-values less than 0.05 were considered statistically significant. *p<0.05; **p<0.01; ***p<0.001.

© 2022 Marino C et al. *JAMA Network Open.*

eFigure 4. Estimates from the cross-lagged model for the reciprocal association between disturbed sleep and depression across adolescence excluding the 10-year-old time point (n=1113).

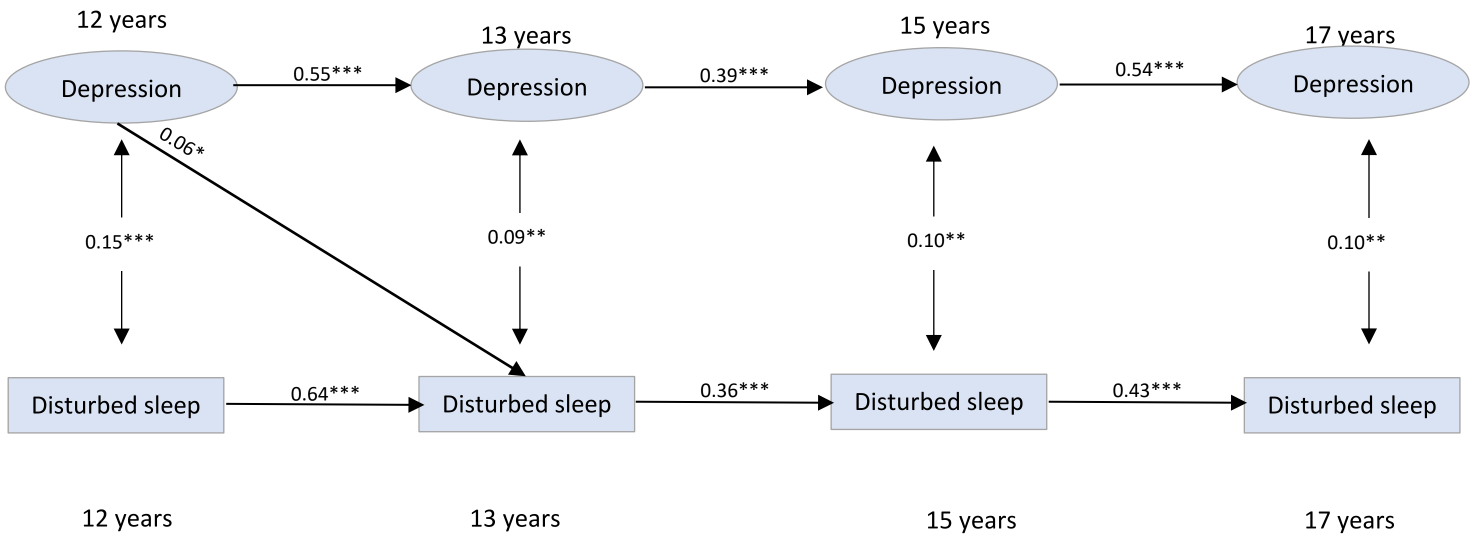

© 2022 Marino C et al. *JAMA Network Open.*

eFigure 4 legend

Source. Data are courtesy of the Quebec Institute of Statistics.
All estimates are standardized. Fit statistics: Chi-square=29.937(18), p<.038; RMSEA=0.024 [0.006, 0.039]; CFI=0.995; TLI=0.988. CFI = comparative fit index; TLI = Tucker Lewis index; RMSEA= root mean square error of approximation. Covariates were socio-economic status and maternal depression. Non-significant paths are not shown.
P-values less than 0.05 were considered statistically significant. *p<0.05; **p<0.01; ***p<0.001.

© 2022 Marino C et al. *JAMA Network Open.*

eFigure 5. Estimates from the cross-lagged model for the reciprocal associations between disturbed sleep and depression from 5 to 17 years of age (n=1085) .

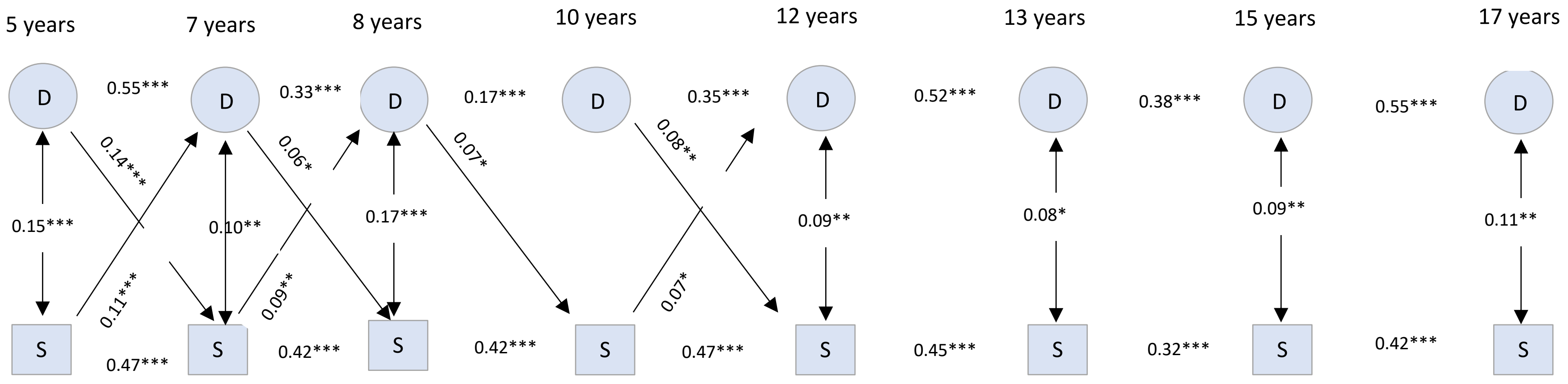

© 2022 Marino C et al. *JAMA Network Open.*

eFigure 5 legend

Source. Data are courtesy of the Quebec Institute of Statistics.
All estimates are standardized. Fit statistics: Chi-square=155.200(88), p<.001; RMSEA=0.027 [0.020, 0.033]; CFI=0.987; TLI=0.978. CFI = comparative fit index; TLI = Tucker Lewis index; RMSEA= root mean square error of approximation. Covariates were socio-economic status and maternal depression. Non-significant paths are not shown.
P-values less than 0.05 were considered statistically significant. *p<0.05; **p<0.01; ***p<0.001.
D=depression
S= disturbed sleep

© 2022 Marino C et al. *JAMA Network Open.*
